# Effect of precooling on the assessment of pain during local anesthesia in adults: A systematic review and meta-analysis 

**DOI:** 10.4317/jced.62351

**Published:** 2025-03-01

**Authors:** Ana Beatriz Leme de Andrade, Sandra Lúcia Dantas de Moraes, Arthur José Barbosa de França, Rayanna Thayse Florêncio Costa, Eduardo Piza Pellizzer, Belmiro Cavalcanti do Egito Vasconcelos

**Affiliations:** 1 MsC student, Department of Oral and Maxillofacial Surgery, Faculty of Dentistry, University of Pernambuco (UPE), Recife, PE, Brazil; 2 Associate Professor, Division of Oral Rehabilitation, Faculty of Dentistry, University of Pernambuco (UPE), Recife, PE, Brazil; 3 PhD student, Department of Oral and Maxillofacial Surgery, Faculty of Dentistry, University of Pernambuco (UPE), Recife, PE, Brazil; 4 PhD student, Division of Oral Rehabilitation, Faculty of Dentistry, University of Pernambuco (UPE), Recife, PE, Brazil; 5 Full Professor, Department of Dental Materials and Prosthodontics, São Paulo State University (UNESP), Araçatuba, SP, Brazil; 6 Associate Professor, Department of Oral and Maxillofacial Surgery, Faculty of Dentistry, University of Pernambuco (UPE), Recife, PE, Brazil

## Abstract

**Background:**

Various methods aim to reduce pain from local anesthesia injections. While commonly used, topical anesthetics have limitations. This systematic review aimed to assess whether precooling is a more effective alternative to topical anesthetics for pain control during local anesthesia injections in adults.

**Material and Methods:**

The review followed PRISMA guidelines and used the PICO framework. The protocol was registered in PROSPERO (CRD42023446314). Independent searches were conducted in PubMed/MEDLINE, SCOPUS, and Web of Science for studies published up to November 2024. Articles meeting the eligibility criteria were included. Data were extracted by one author and verified by another. A meta-analysis was performed to evaluate pain between the precooling and topical anesthetic groups after anesthesia and during needle insertion.

**Results:**

Eight randomized clinical trials met the inclusion criteria, with a combined sample of 415 patients. Six of the eight studies reported lower pain scores in patients who received precooling compared to those treated with topical anesthetics. Various agents were used for both local cooling and topical anesthesia. Meta-analysis showed statistically significant pain reduction in favor of precooling after anesthesia; however, no significant differences were found during needle insertion.

**Conclusions:**

Precooling may be an effective alternative to topical anesthetics, reducing pain associated with needle insertion and anesthetic injection in adults. Further research is warranted to establish a standardized application protocol.

** Key words:**Local anesthesia, pain, precooling, cryoanesthesia, systematic review.

## Introduction

Pain is a complex sensory experience involving cognitive and affective components (1). The control of pain has always been a concern in dentistry (2), especially during the injection of local anesthetics, which can generate fear and anxiety in some individuals during dental procedures (3).

Distraction techniques, anesthetic buffering, adjacent tissue vibration, the application of heat/cold, and the administration of topical anesthetics are some of the methods used to reduce pain related to local anesthesia (4). Topical anesthetics are widely used but have some disadvantages, such as the application time, unpleasant flavor (5), and the possibility of causing an allergic reaction (6).

Precooling consists of the application of cold to a specific part of the body, affecting all cells in the region with the aim of interrupting the local nerve conduction of pain impulses. This technique can be performed using a freeze spray or ice (7) and constitutes an auxiliary tool in treatment and recovery in the health field due to its effectiveness, low cost, and portability (8).

Previous studies have investigated the benefits of precooling in pediatric patients (9,10); However, pain perception in children differs from that in adults due to the enormous physiological and psychological variability throughout the entire age spectrum of the pediatric population (11).

Although many clinical trials have been conducted on this topic, no systematic reviews have been conducted comparing the use of precooling to topical anesthetics prior to local anesthesia in adults. Therefore, the aim of the present systematic review was to determine whether precooling is a better alternative to substances currently used for topical anesthesia in the control of pain during the injection of local anesthesia in adults.

## Material and Methods

-Protocol and Registration

The present study adhered to the Enhancing the Quality and Transparency of Health Research (EQUATOR network) recommendations, including the Preferred Reporting Items for Systematic Reviews and Meta-Analyses (i.e., PRISMA) (12,13); The review protocol was registered in the PROSPERO database (CRD42023446314).

-Focused question

Based on the PICO strategy—population (adult patients submitted to dental procedures under local anesthesia); intervention (use of precooling); comparator (use of topical anesthetics: lidocaine, benzocaine, EMLA); and outcome (pain)—the following focused question was proposed: “Does the use of precooling lead to less pain during needle insertion and the injection of local anesthesia in dental procedures in adults?”

-Study selection criteria

Studies eligible for inclusion were randomized clinical trials (RCTs) and controlled clinical trials (CCTs) comparing the use of precooling and topical anesthetics prior to the administration of local anesthesia in dental procedures performed on adults (people aged 18 and over), evaluating patients ‘ pain during needle insertion or pain after injection using the Visual Analogue Scale (VAS) or Numerical Rating Scale (NRS). No restrictions were imposed with regards to language or year of publication.

The exclusion criteria were: Studies with participants under 18 years of age, studies with no description of the precooling or topical anesthetic or dental procedures performed, trials in which the outcomes of interest were not available for analysis and the original values could not be retrieved after contacting the authors, and studies for which the full-text article was unavailable.

-Search strategy

A search was conducted independently by two authors (A.B.L.A and A.J.B.F) in PubMed/MEDLINE, SCOPUS, and Web of Science databases. The search was last updated on 11/05/2024 using the following terms: (precooling OR pre-cooling OR cooling OR ice OR cryotherapy OR refrigerant) AND (anesthesia OR local anesthesia OR injection OR nerve block OR nerve blockade) AND (dental OR tooth OR teeth). A manual search was performed by the same authors for articles published in the following journals from January 2019 to November 2024: International Journal of Oral and Maxillofacial Surgery; Journal of Oral and Maxillofacial Surgery; Journal of Cranio-Maxillofacial Surgery; British Journal of Oral and Maxillofacial Surgery; and Clinical Oral Investigations. The search was also performed from ClinicalTrials.gov and the reference lists of the selected articles (gray literature). The data are summarized in  Table 1.

-Study selection process

**Figure 1:**
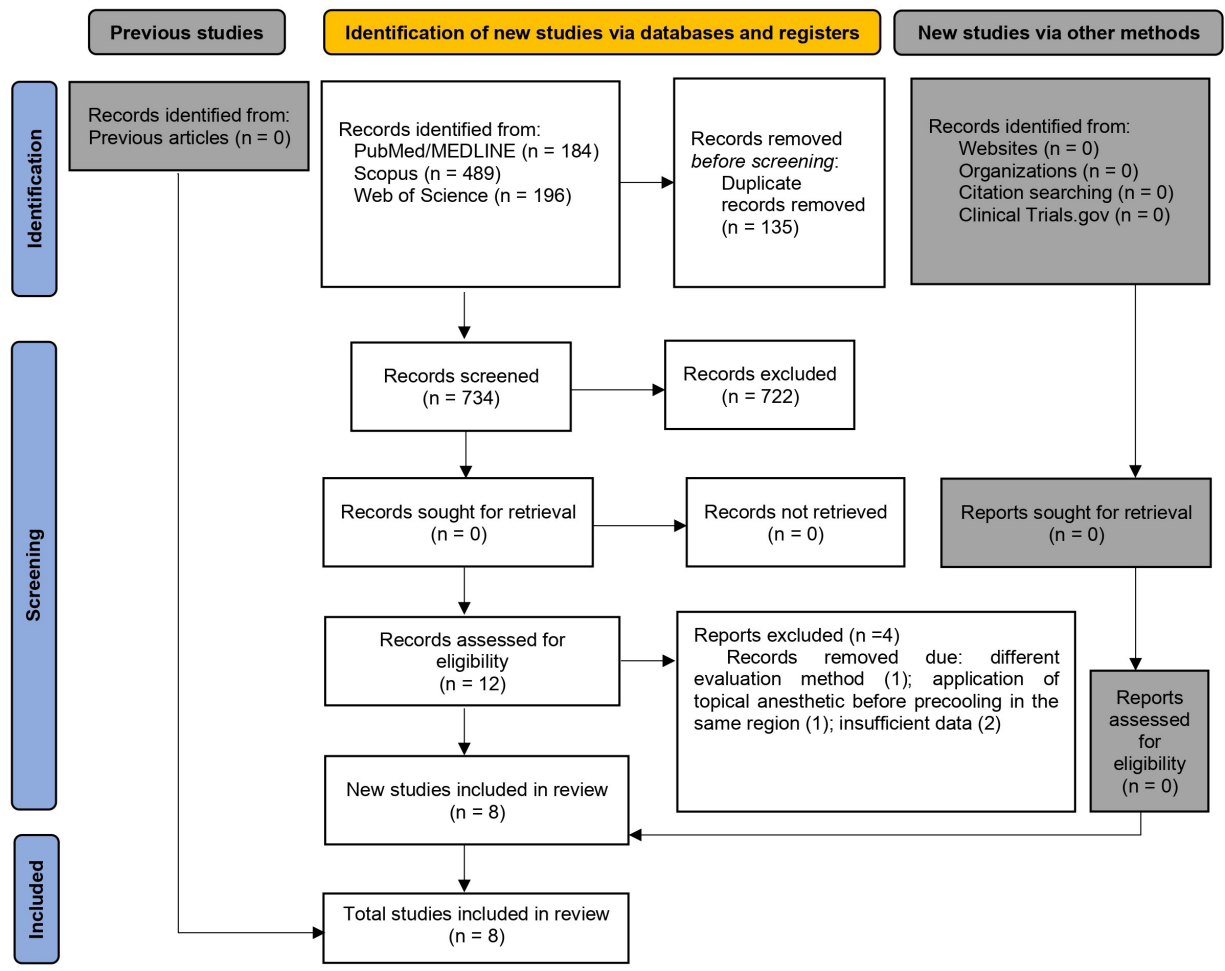
Flow diagram describing the studies selection.

The records retrieved from the databases were imported to the EndNote reference manager for the identification and removal of duplicates, as illustrated in the PRISMA flowchart (Fig. 1). The selection process was conducted in two phases. In phase 1, two researchers (A.B.L.A. and A.J.B.F.) independently examined the titles and abstracts of all records, applying the eligibility criteria (blind process) for the preselection of articles for further analysis. In phase 2, the same two reviewers independently applied the eligibility criteria to the full text of the preselected articles (blind process).

-Data extraction and synthesis process

The following data were collected from the articles included in the review: study design; age of participants and sample size; type of local anesthetic; type of topical anesthetic; type of precooling agent; application time (Precooling/Topical anesthesia); application site; procedure performed; pain during needle insertion or pain after injection. Inter-reviewer reliability in the study selection process was calculated using Cohen’s Kappa statistic, assuming an acceptable threshold of 0.8. Divergences of opinion in any stage were resolved by discussion and mutual consensus with a third reviewer (B.C.E.V.). The final decision or selection was always based on a review of the full text.

-Risk of bias and quality assessment

The studies were independently evaluated by two authors (A.B.L.A and A.J.B.F) using specific risk of bias and methodological quality assessment tool for RCTs (ROB 2). Agreement was reached in a consensus meeting with a third reviewer (B.C.E.V.), as needed.

-Grading the body of evidence

Quality of evidence was assessed using the Grading of Recommendations Assessment, Development, and Evaluation (i.e., GRADE) system. This system classifies the quality of evidence as high, moderate, low, or very low based on factors that consider study design, risk of bias in reported outcomes, inconsistency of outcomes among studies, indirectness of reported outcomes, imprecision of reported outcomes, and potential publication bias. The strength of the recommendation was graded as strong or weak (13,14).

-Data extraction and synthesis

Full-text articles of the included studies were analyzed. Descriptive results were presented in the form of text, Figures, and Tables, in accordance with the PICO strategy. If the selected studies reported multiple intervention groups, only the groups that met the selection criteria were included. Quantitative measures were described as absolute frequencies and means ± standard deviation. In cases of missing data or data available only in graph form, the corresponding authors were contacted via e-mail and/or social media, when necessary, to obtain details on the study design and data clarification. Data available only in graph form were extracted using WebPlotDigitizer version 4.4, if necessary.

-Summary measures and synthesis of the results

Descriptive data were stratified according to the study design. The included studies were classified according to the Oxford Center for Evidence-Based Medicine (15) and summarized in hierarchical categories of the pyramid of levels of evidence (16). Qualitative analyses were presented in the form of text, graphs, and Tables, in accordance with the PICO strategy.

The meta-analysis (Reviewer Manager 5 software, Cochrane Group) was performed for the outcomes “pain during needle insertion”, and “pain after injection”. The data were evaluated using Mantel-Haenszel and/or inversion of variance tests and continuous data (mean and standard deviation) evaluated with 95% confidence intervals. MD (mean difference) values were considered statistically significant at *P* < .05. In addition, the I² values were used to express the percentage of heterogeneity, with data with 25% corresponding to low heterogeneity, 50% moderate heterogeneity, and 75% high heterogeneity. A random-effects model was adopted.

## Results

-Study selection

The search of the databases led to the retrieval of 869 records: 184 from MEDLINE/PubMed, 196 from Web of Science, and 489 from Scopus. The level of agreement between the reviewers was high (K > 80%). No additional studies were found through the lists of the articles retrieved or the search of protocol registers or other sources. After the removal of duplicates (n= 135), the titles and abstracts of 734 articles were screened. Twelve articles were submitted to full-text analysis, four of which were excluded for the following reasons: different pain assessment method (n = 1) (17), application of topical anesthetic before precooling in the same region (n=1) (18) and incomplete data (n=2) (19,20). Thus, eight randomized clinical trials were included in the review. The flowchart of the article selection process in accordance with the PRISMA statement is displayed in Fig. 1.

-Study characteristics

 Table 2 displays the characteristics of the eight randomized clinical trials, such as study design, age group, sample size, type of anesthetic, needle gauge, cooling agent, cooling time, topical anesthetic, time of action of topical anesthetic, procedure performed, and region submitted to anesthesia. Five of the eight articles reported the local anesthesia used in the clinical trial (5,8,21-23).

Lidocaine 2% with different concentrations of epinephrin (1:80,000; 1:100,000) was the most used local anesthesia. Needle gauge ranged from 25 to 30 G. One of the studies failed to report the gauge of the needle used in the injection (6). The clinical procedures to which the participants were submitted were extractions, restorative procedures, and periodontal treatment, involving anesthesia administered to the maxillary or mandibular region.

Pain during the insertion of the needle and after the injection of the anesthetic was assessed using numerical classification scales (Visual Analogue Scale (VAS) -0 to 10 cm- and Numerical Rating Scale (NRS) - 0 to 10- ( Table 3). Two studies (8,23) found a better result for the topical anesthetic (EMLA) compared to precooling for patient-reported pain, whereas the other articles described results favoring precooling.

-Quality assessment of the studies

Cochrane Risk-of-Bias Tool for Randomized Trials

**Figure 2:**
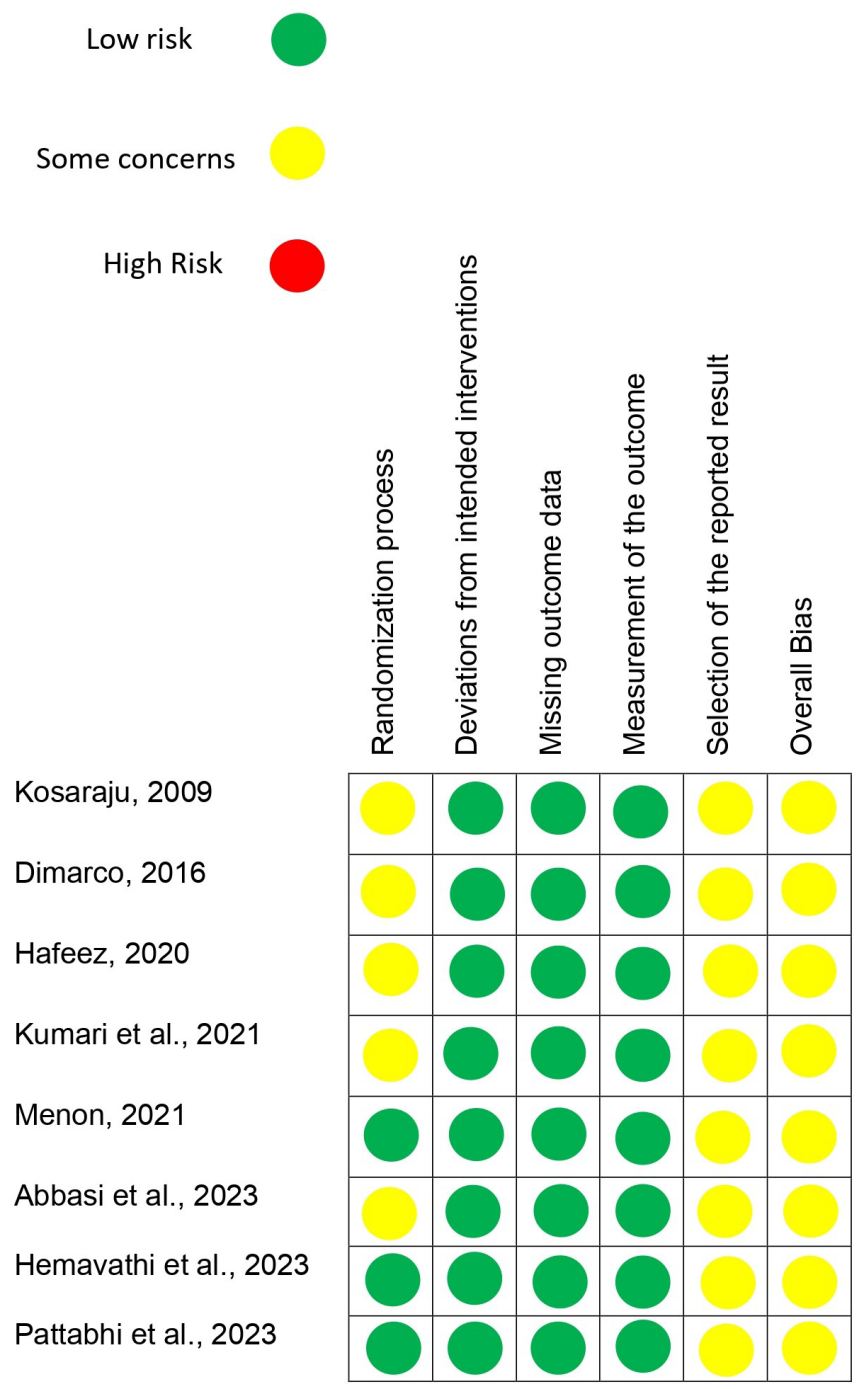
Assessment of the risk of bias in the included studies based on the Cochrane Risk of Bias Tools (ROB 2.0). High risk of bias (red); Some concerrns (yellow); Low risk of bias (green).

Moderate risk bias was found for randomization process in five studies (4-6,21,22), except in three (3,8,23) in which low risk of bias was found. A low risk of bias was found in all studies regarding deviation from the intended intervention, missing outcome data, and measurement of the outcomes. Uncertain risk was observed in all studies concerning the selection of the reported result. Based on these findings, the studies had a moderate overall risk of bias (Fig. 2).

-Meta-analysis

Pain during needle insertion – Precooling vs. Topical Anesthetic

**Figure 3:**
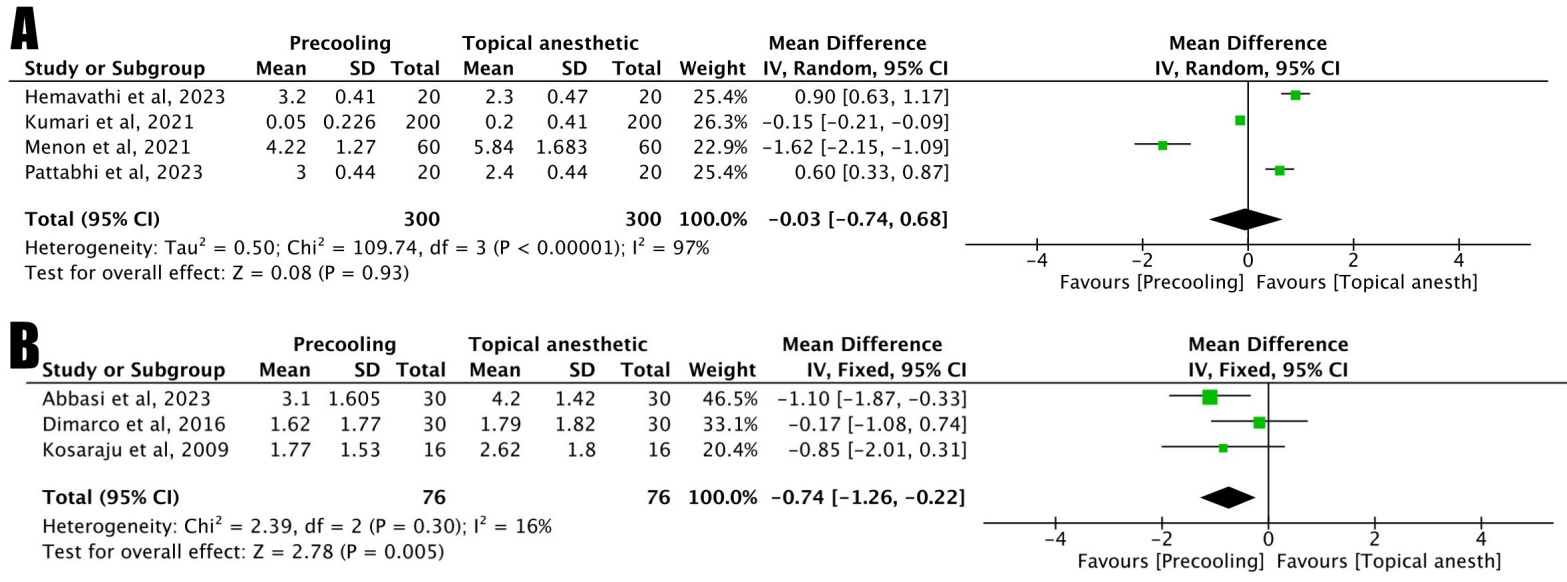
Meta-analysis - Pain and needle insertion. A) Pain during needle insertion – Precooling vs. Topical Anesthetic (Forest plot); B) Pain after needle insertion – Precooling vs. Topical Anesthetic (Forest plot).

Four studies compared precooling and topical anesthetic protocols for pain during needle insertion. In the meta-analysis using a random-effects model, no statistically significant difference was found between the two methods (*p* = 0.93; MD: -0.03; CI: -0.74 to 0.68). Heterogeneity among the studies was high (χ²: 109.74; I² = 97%; *p* <0.00001) (Fig. 3A). One study was not included in the meta-analysis because it did not present the standard deviation (6).

Pain after injection – Precooling vs. Topical Anesthetic

Three studies compared precooling and topical anesthetic protocols for pain after anesthesia. In the meta-analysis using a random-effects model, a statistically significant difference was found between the two methods (*p* = 0.005; MD: -0.74; CI: -1.26 to - 0.22). Heterogeneity among the studies was low (χ²: 2.39; I² = 16%; *p* = 0.30) (Fig. 3B).

-Grade

Comparing precooling and topical anesthetic, the quality of evidence was very low for pain during needle insertion and low for pain after the administration of anesthesia due to inconsistency and the limitations of the study design. Comparing the same groups, the results were low due to the limitations of the study design and substantial heterogeneity (*p* <0.00001, I² = 98% and *p* = 0 .20, I² = 39%, respectively). The small sample size also exerted an influence on the low quality of the evidence ( Table 4).

## Discussion

The studies included in the present systematic review demonstrated significant differences in pain during needle insertion and after injection of the anesthesia when comparing patients (adults) submitted to pre-anesthetic cooling and those who received a topical anesthetic prior to needle insertion. Overall, the studies had a moderate risk of bias.

**Figure 4:**
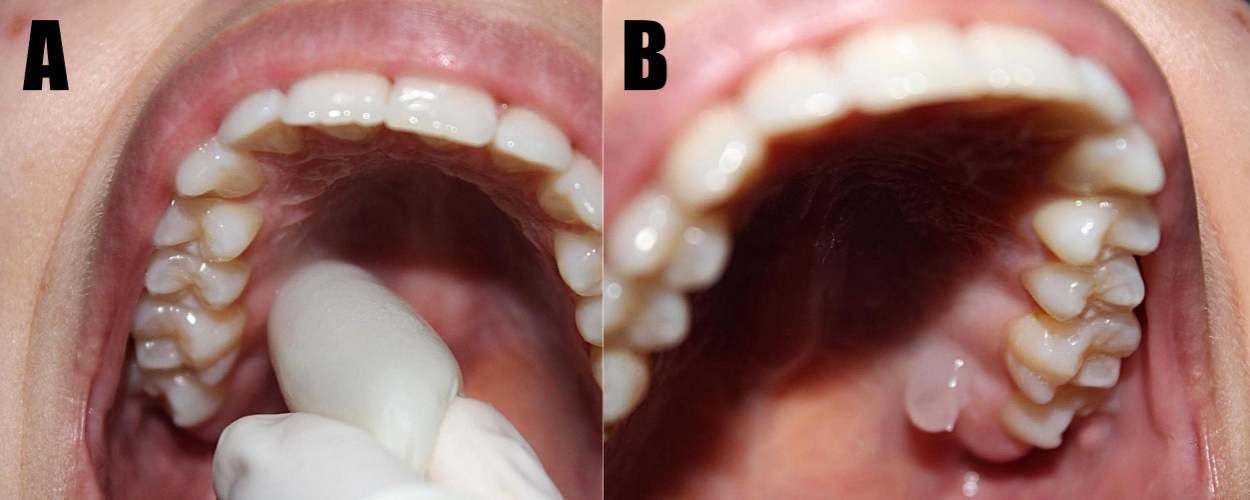
Illustration of different techniques. A) Precooling with ice before local anesthetic infiltration . B) Topical anesthetic in the palatal region before infiltration with local anesthetic.

Some limitations were observed, such as a lack of standardization with regards to the application time of the cooling agent (5 seconds, 30 seconds, 1 minute and 2 minutes) and topical anesthetic (15 seconds, 30 seconds and 2 minutes), type of topical anesthetic (benzocaine 20%, lidocaine spray 10%, lidocaine gel 2%, lidocaine gel 5%, and EMLA 5%), and cooling agent employed (refrigerant - tetrafluoroethane, ethyl chloride, pentafluoroethane -, refrigerated anesthetic cartridge, and ice), needle gauge (25 G, 27 G and 30 G) and procedures to which the participants were submitted (periodontal treatment, extraction, endodontic treatment, and restorative treatment) ( Table 2). Figure 4 demonstrate the application of precooling (A) and intraoral topical anesthesia (B), respectively.

Local anesthesia is considered the most painful step of small procedures and is associated with the induction of fear and anxiety in patients (3,22). Among local anesthetics, lidocaine is considered one of the most widely used substances for the achievement of painless dental treatment. Its use was approved in 1948 and it is sold with or without a vasoconstrictor (epinephrin) in different concentrations (22,24). Among the eight studies included in the present systematic review, five described the local anesthetic used, with lidocaine 2% the anesthetic of choice in five studies combined with different concentrations of epinephrin 1:80,000 (8,22,23) and 1:100.000 (5,21) ( Table 2).

Different methods have been used to minimize pain complaints related to the injection of local anesthesia, such as distraction techniques, anesthetic buffering, adjacent tissue vibration, the application of heat/cold, and the application of topical anesthetics (4). The application of a topical anesthetic in the region to be anesthetized is routine practice in dentistry for reducing pain. However, the disadvantages of this method include the longer application time and unpleasant taste (5) as well as the possibility of provoking an allergic reaction (6,20). Different topical anesthetics are employed, the most widely used of which are benzocaine 20% and lidocaine. Benzocaine is an ester-based agent capable of producing an effect in 30 seconds, but requiring two to three minutes to reach an adequate depth and intensity (25).

Lidocaine can be used both as a topical anesthetic and injectable, the former of which can be in the form of gels and sprays in different concentrations. In a study developed by Garg *et al*. (2016) (26), benzocaine 20% and lidocaine gel 2% were equally effective at reducing pain related to needle insertion and both substances achieved better results compared to a placebo. Nair and Gurunathan (2019) (27) reported similar findings, with no significant difference between the two substances in terms of anesthetic efficacy. Among the eight studies included in this review, two employed benzocaine 20%, two used lidocaine gel 2%, one used lidocaine gel 5%, one used lidocaine gel 10%, and two used EMLA 5% ( Table 2).

Although some studies have employed EMLA as a topical anesthetic, this mixture was originally developed by the manufacturer for application to intact skin. EMLA is a eutectic mixture of prilocaine and lidocaine at a 1:1 proportion, with the recommendation of use one hour prior to the procedure to be performed due to the need to pass through the intact barrier (24). The application of this substance to mucosa was performed for two minutes, achieving good results with regards to the reduction in pain (8).

Precooling consists of the application of cold to a specific part of the body, affecting all cells in the region, with the aim of interrupting the local nerve conduction of pain impulses and is performed with freeze sprays or the use of ice (7). Several researchers have compared the pain reduction capacity of an intraoral injection of commercial topical anesthetics to local cooling to determine an effective method that offers greater safety to patients. In four studies, a refrigerant agent was used for cooling (3,5,21,22), whereas ice was the option adopted in four clinical trials (4,6,8,23), which was applied for different periods of time ( Table 2).

Studies have suggested that patients submitted to local cooling with ice have a lower pain intensity compared to those who receive a refrigerant, explaining this difference by the shorter contact time with the refrigerant in the individual (7,28). Among the trials analyzed in the present review, no comparison was made between ice and a refrigerant. Therefore, this difference could not be verified. The application time of refrigerant agents was five seconds (3,5,21) and 30 seconds (22), whereas ice was applied for 1 minute (6) and 2 minutes (4,8,23), with no comparisons between the types of refrigerants and no definition of the best application time ( Table 3).

The gauge of the needle used for the injection of the local anesthetic is an important factor to consider in the assessment of pain complaints by patients. A study comparing two needle gauges for intrapulpal injection with the use of topical anesthesia as an adjuvant (29), found that patients having received the local anesthetic through a 31 G needle with or without the topical anesthetic reported less pain intensity compared to those who received the local anesthetic through a 27 G needle. In contrast, Hussain *et al*. (2020) (30) found no significant difference in pain reported by patients during the administration of anesthetic using needles with gauges of 23 G and 27 G.

The analysis of pain in patients submitted to precooling and those for whom a topical anesthetic was used prior to needle insertion revealed results favoring precooling in six of the eight studies, whereas Hemavathi *et al*. (2023) and Pattabhi *et al*. (2023) (8,23), found that the participants reported a more effective reduction in pain when submitted to the application of a local anesthetic (EMLA). The insertion of the needle was performed in the maxilla or mandible, depending on the region in which the procedure was to be performed. The anesthetic technique was performed in the maxilla in four studies (4,5,8,21), while the anesthesia was performed in the mandibular region in two studies (3,22,23). One study did not specify the technique used (6). The studies included in this review did not aim to compare complaints of pain during injection in different intraoral sites.

The selected studies evaluated the patient’s pain using the Visual Analogue Scale (VAS) or the Numerical Rating Scale (NRS), as they are similar scales and easy to interpret. The VAS is typically a horizontal line, 100 mm (10 cm) in length, anchored by word descriptors at each end. The patient marks the point on the line that best represents their perception of the current state. The VAS score is determined by measuring in millimeters from the left-most end of the line to the point marked by the patient (31). The Numeric Rating Scale (NRS) is similar to the VAS, with the left end labeled ‘no pain’ and the right end labeled ‘worst pain imaginable’ (or something similar). The key difference is that, instead of an unmarked line, numbers from 0 to 10 are evenly spaced across the scale (32). Of the eight studies included in the review, six used the VAS to assess pain (3,5,8,21–23), while two used the NRS (4,6). To facilitate interpretation, the VAS values (100 mm) from the studies were converted to cm ( Table 3).

According to the results of the meta-analysis of this study, when evaluating pain between the precooling and topical anesthetic groups after anesthesia, statistically significant improvements were observed in favor of precooling (*p*= 0.005); In the evaluation between the same groups during needle insertion, there were no statistically significant differences (*p*= 0.93).

Based on the present findings, precooling is a viable option for minimizing pain during the application of local anesthesia, offering safety, effectiveness, and low cost. Considering the heterogeneity among the studies analyzed, further randomized clinical with well-defined methods should be conducted to establish a precooling protocol and enable a better quality of evidence on this topic.

## Figures and Tables

**Table 1:** Search strategy for each database and journals.

Database	Search strategy	Filter
PubMed/MEDLI NE	(precooling OR pre-cooling OR cooling OR ice OR cryotherapy OR refrigerant) AND (anesthesia OR local anesthesia OR injection OR nerve block OR nerve blockade) AND (dental OR tooth OR teeth)	No filters applied
Scopus	"precooling" OR "pre-cooling" OR "cooling" OR "ice" OR "cryotherapy" OR "refrigerant" AND "anesthesia" OR "local anesthesia" OR "injection" OR "nerve block" OR "nerve blockade" AND "dental" OR "tooth" OR "teeth"	Document type: article; Subject area: Dentistry Source type: Journal
Web of Science	(precooling OR pre-cooling OR cooling OR ice OR cryotherapy OR refrigerant) AND (anesthesia OR local anesthesia OR injection OR nerve block OR nerve blockade) AND (dental OR tooth OR teeth)	No filters applied
Clinical Trials.gov	precooling OR pre-cooling OR cooling OR ice OR cryotherapy OR refrigerant	Status: completed; Studies: with results; Study type: interventional (clinical trial)
Journals	Search strategy: Last 5 years	
International Journal of Oral and Maxillofacial Surgery; Journal of Oral and Maxillofacial Surgery; Journal of Cranio-Maxillofacial Surgery; British Journal of Oral and Maxillofacial Surgery; and Clinical Oral Investigations		

**Table 2:** General Data.

Author, year	Design	Age/Sample	Local Anesthetic	Precooling /Time	Topical Anesthetic /Time	Needle gauge	Procedure performed	Anesthesia technique
Kosaraju 2009	RCT / SM	19 – 65 / 16	Lidocaine 2% + ep. 1:100.000	Refrigerant agent / 5 secs.	Benzocaine 20% / 2 min.	30G	Periodontal treatment	GPNB
Dimarco 2016	RCT / SM	19 – 77 / 30	Lidocaine 2% + ep. 1:100.000	Refrigerant agent / 5 secs.	Benzocaine 20% / 2 min	27G	Restoration	AMSA
Hafeez, 2020	RCT / SM	20 – 60 / 9	NR	Ice /1 min.	Lidocaine spray 10% / 15 secs.	NR	Extraction	NR
Kumari et al., 2021	RCT / SM	18 – 60 / 200	NR	Ice / 2 min.	Lidocaine gel 2% / 2 min.	25G	Extraction	GPNB
Menon 2021	RCT / SM	> 18 / 60	NR	Refrigerant agent / 5 secs.	Lidocaine gel 2% / 2 min.	30G	Extraction	IANB
Abbasi et al., 2023	RCT	20 – 40 / 60	Lidocaine 2% + ep. 1:80.000	Refrigerant agent / 30 secs.	Lidocaine gel 5% / 30 secs.	27G	Extraction, endodontics, and restoration	BNB
Hemavathi et al., 2023	RCT / SM	> 18 / 20	Lidocaine 2% + ep. 1:80.000	Ice / 2 min.	EMLA 5% /2 min.	25G	Extraction	GPNB
Pattabhi et al., 2023	RCT / SM	> 18/ 20	Lidocaine 2% + ep. 1:80.000	Ice/ 2 min	EMLA 5%// 2min	25G	Extraction	BNB

RCT: Randomized clinical trial, %: percent, ep: epinephrine, NR: not reported, min.: minutes, secs.: seconds, G: gauge, AMSA: Anterior middle superior alveolar, IANB: Inferior alveolar nerve block, MSA: Middle superior alveolar, BNB: Buccal nerve block, GPNB: Greater palatine nerve block, PSA: Posterior alveolar superior; SM: Split-mouth.

**Table 3:** Pain Data.

Author, year	Pain during needle insertion		Pain after injection		M ± SD	
Precooling	Precooling	Topical anesthetic	Precooling	Topical anesthetic	Precooling	Topical anesthetic
Kosaraju 2009	-	-	1.77	2.62	1.77 ± 1.53 cm	2.62 ± 1.80 cm
Dimarco 2016	-	-	1.62	1.79	1.62± 1.77 cm	1.79 ± 1.82 cm
Hafeez 2020*	2.13	4.25	-	-	2.11 (M)	4.11 (M)
Kumari et al., 2021*	0.05	0.20	-	-	0.05 ± 0.226	0.2 ± 0.41
Menon 2021	4.22	5.84	-	-	4.22 ± 1.27 cm	5.84 ± 1.683 cm
Abbasi et al., 2023	-	-	3.10	4.2	3.10 ± 1.605 cm	4.2 ± 1.42 cm
Hemavathi et al., 2023	3.2	2.3	-	-	3.2 ± 0.41 cm	2.3 ± 0.47 cm
Pattabhi et al., 2023	3.0	2.4	-	-	3.0 ± 0.44 cm	2.4 ± 0.44 cm
According to the Visual Analogic Scale (VAS 0-10cm), *According to the Numerical Rating Scale (NRS 0-10)						

**Table 4:** Evidence certainty assessment with GRADE (Classification of Recommendations, Assessment, Development and Evaluation).

Question: Precooling compared to Topical Anesthetic for Patients undergoing local anesthesia in dental procedures											
Certainty assessment							Nº patients		Effect		Certainty
Nº of studies	Study desing	Risk of bias	Inconsistency	Indirectness	Imprecision	Other considerations	Precooling	Topical Anesthetic	Relative(95% CI)	Absolute(95% CI)
Pain during needle insertion (assessed with: VAS (3,8,23), NRS (4); Scale from: 0 to 10)											
4	randomized trials	not serious	very serious ^a^	not serious	serious ^b^	none	300	300	-	0 (0 to 0)	ѳοοο Very Low
Pain after anesthesia (assessed with: VAS (5,21,22); Scale from: 0 to 10)											
3	randomized trials	not serious	serious ^a^	not serious	serious ^b^	none	136	136	-	0 (0 to 0)	ѳѳοο Low
CI: Confidence interval; VAS: Visual Analogue Scale.; NRS: Numerical Rating Scale											

Explanations a. Due to presence of substantial heterogeneity (*p*<0.00001, I²= 98%). b. Due to small sample size c. Due to presence of substantial heterogeneity (*p*=0.20, I²= 39%).

## Data Availability

The datasets used and/or analyzed during the current study are available from the corresponding author.
